# The Mediating Role of Workplace Social Capital and Affective Occupational Commitment in the Relationship Between In‐Group/Out‐Group Dynamics and Work Improvement and Innovation Among Nurses: A Longitudinal Study

**DOI:** 10.1155/jonm/4125257

**Published:** 2026-10-22

**Authors:** Chen Huang, Yu Yang, Yifan Wu, Zuoming Zhang, Hongmei Liu, Lin Cai, Shuang Zang

**Affiliations:** ^1^ Department of Nursing The Second Affiliated Hospital of Soochow University Suzhou China suda.edu.cn; ^2^ Department of Community Nursing School of Nursing China Medical University Shenyang China cmu.edu.tw; ^3^ Department of Vascular Surgery The First Hospital of China Medical University Shenyang China cmu.edu.cn; ^4^ Xiangya School of Nursing Central South University Changsha China csu.edu.cn; ^5^ Department of Nursing Baoding Fourth Central Hospital Baoding China; ^6^ School of Education Sichuan Institute of Industrial Technology Deyang China

**Keywords:** affective occupational commitment, in-group/out-group dynamics, nurses, work improvement and innovation, workplace social capital

## Abstract

**Background:**

Work improvement and innovation among nurses is crucial for optimizing nursing processes and enhancing service quality. However, the relationship between in‐group/out‐group dynamics and work improvement and innovation, as well as longitudinal mechanisms, remains unclear.

**Objective:**

To investigate the relationship between in‐group/out‐group dynamics and work improvement and innovation among nurses within a longitudinal research framework, while exploring the mediating roles of workplace social capital and affective occupational commitment.

**Methods:**

Using a three‐wave longitudinal design, 436 nurses from Baoding Fourth Central Hospital were surveyed in stages via an online platform in March 2024 (T1), June 13–July 12, 2024 (T2), and October 2024 (T3). Specifically, in‐group/out‐group dynamics were measured at T1, workplace social capital and affective occupational commitment at both T1 and T2, and work improvement and innovation at both T1 and T3. Data were analyzed using SPSS 29.0 for descriptive statistics and Pearson’s correlation analysis, and Mplus 8.0 for structural equation modeling.

**Results:**

All study variables were significantly correlated (*p* < 0.001). In‐group/out‐group dynamics (T1) among nurses were negatively associated with work improvement and innovation (T3) both directly and indirectly through workplace social capital (T2) and affective occupational commitment (T2). The total indirect effect accounted for 39.19% of the total effect.

**Conclusions:**

From a longitudinal perspective, this study reveals the mechanisms by which in‐group/out‐group dynamics impede work improvement and innovation through workplace social capital and affective occupational commitment among nurses. This study presents new empirical evidence for understanding the evolution of improvement and innovation in nursing practice, providing valuable implications for nursing management.

**Implications for Nursing Management:**

Nursing managers should mitigate in‐group/out‐group dynamics among nurses to foster workplace social capital and strengthen affective occupational commitment, thereby effectively promoting nurses’ work improvement and innovation.

## 1. Introduction

In the field of nursing, nurses’ improvement and innovation serve as an important driving force for advancing the nursing profession and improving the quality of care [1]. The World Health Organization has stated that nurses, as the primary workforce within the healthcare system, deliver up to 80% of primary healthcare services and that their innovative capacity is essential in enhancing healthcare productivity [2]. Multiple studies have shown that nurses’ innovative work behaviors are closely associated with nursing quality and organizational performance [3, 4]. Work improvement and innovation is defined as the intentional creation, introduction, and application of novel ideas by employees within their work, teams, or organizations to enhance individual or organizational performance [5]. In this study, work improvement and innovation specifically emphasize nurses exploring and developing new technologies or methods in clinical practice, optimizing workflows, and driving implementation to promote health, prevent illness, and elevate the quality of care [6, 7]. As frontline providers of healthcare services and implementers of health policies, nurses are uniquely positioned to identify deficiencies, gaps, and challenges within healthcare systems across diverse settings [8]. However, Zhang et al. [9] and Fu et al. [10] reported that innovation behaviors among nurses in China remained at a moderately low level, failing to meet the rapidly evolving demands of the nursing profession. Consequently, promoting nurses’ active engagement in work improvement and innovation represents a crucial strategy for enhancing the quality of nursing services.

Organizational management factors are indispensable for promoting improvement and innovation in nursing practice. Existing research indicates that the organizational climate, as a crucial contextual element, has considerable theoretical value for understanding employees’ innovative behavior [11, 12]. Current studies primarily focus on innovative and supportive organizational climates, often implicitly assuming relatively equitable organizational support among members, while neglecting individuals’ perceptions of differential treatment within organizations. In‐group/out‐group dynamics can be conceptualized through the metaphor of ripples on water, whereby interpersonal relationships become progressively weaker with increasing relational distance, accompanied by reduced access to resources and power [13]. In‐group/out‐group dynamics reflect both the relational distance between organizational members and resource allocators and employees’ perceptions of unequal resource distribution [14]. Within nursing teams, such perceptions manifest as perceived differential treatment, hierarchical authority, and relationship‐oriented interactions [15, 16]. Previous research has indicated that stronger in‐group/out‐group dynamics are associated with lower team cohesion, reduced knowledge sharing and collaborative efforts, and lower individual creativity [17]. However, current research has paid limited attention to the subjective cognitive processes underlying the association between in‐group/out‐group dynamics and work improvement and innovation, and the potential mechanisms underlying this relationship remain insufficiently understood. Therefore, longitudinal investigation is necessary to explore the pathways linking in‐group/out‐group dynamics with nurses’ work improvement and innovation in order to promote the sustainable development of nursing services.

Previous research has examined the impact of in‐group/out‐group dynamics on individual organizational behavior through the lens of trait activation theory [15]. The theory posits that individual traits influence specific behaviors only when activated by particular situations [18]. However, this perspective may not fully capture how nurses cognitively interpret and construct meaning from the social signals of in‐group/out‐group dynamics. According to social information processing theory, individuals’ attitudes, cognitions, and behaviors are constructed through the processing of available social information within their surrounding environment [19]. This theory elucidates the interplay between organizational cues, workplace contexts, and personal beliefs in shaping cognition‐driven behaviors. Research indicates that nurses’ subjective perceptions can accurately predict their behavioral outcomes [20, 21]. Notably, the in‐group/out‐group dynamics examined in this study emphasize nurses’ perceptions of the organizational atmosphere within their workplace rather than the objectively existing organizational environment. Perceived organizational injustice drives nurses to adopt behavioral strategies oriented toward risk aversion and self‐protection, reducing their intrinsic motivation [22], thereby discouraging them from proposing work improvement suggestions or participating in innovative activities. Therefore, in‐group/out‐group dynamics may shape nurses’ negative environmental cognition, ultimately inhibiting their efforts toward work improvement and innovation.

Previous research indicates that workplace social capital among nurses comprises relational networks and social assets characterized by trust, reciprocity, shared understanding, and collaborative interactions [23]. Therefore, within nursing organizations, the social signals transmitted by in‐group/out‐group dynamics may influence not only nurses’ individual perceptions but also their patterns of social interaction and relational network structures. Based on social information processing theory, when nurses perceive hierarchy and inequitable resource distribution within an organization, they may interpret these cues as signs of organizational dysfunction or management failure. This can lead to reduced investment in collegial relationships and weaker development of trust and reciprocity [24], ultimately impeding workplace social capital. The impact of in‐group/out‐group dynamics on workplace social capital among nurses may not occur immediately but may accumulate over time. In addition, a prior study conceptualized the internal dimension of workplace social capital in terms of information sharing, shared vision, and trust among team members [25], which can facilitate knowledge combination and support innovative behavior [26]. Existing research indicates that higher levels of social capital can enhance knowledge sharing [27], collaborative problem‐solving, and cross‐functional support among employees [28], thereby driving improvements in work processes and innovation. Conversely, impaired workplace social capital can result in restricted information exchange and decreased interdepartmental collaboration, potentially constraining the generation and implementation of ideas for improvement and innovation. Thus, workplace social capital may serve as a mediator in the relationship between in‐group/out‐group dynamics and work improvement and innovation among nurses.

Individual‐level emotional attitudes may also play a mediating role in the relationship between in‐group/out‐group dynamics and work improvement and innovation. Affective occupational commitment refers to employees’ emotional attachment, identification, and engagement with the profession [29]. Social information processing theory posits that individuals form and adjust their emotional evaluation and professional attitudes in response to environmental cues [19]. In nursing settings, perceived organizational support and justice climate have been identified as crucial social informational resources associated with affective occupational commitment [30, 31]. However, multiple studies indicate that perceptions of organizational unfairness and unequal resource distribution can negatively impact nurses’ affective commitment and work engagement [32, 33]. The signals of differential treatment conveyed by in‐group/out‐group dynamics may therefore be particularly salient. Repeated exposure to such negative cues during organizational interactions may undermine nurses’ trust in organizational fairness and, in turn, be associated with lower affective occupational commitment. Furthermore, previous research has shown that higher levels of affective occupational commitment can predict more favorable organizational performance [34], which may encourage nurses to proactively propose feasible improvement suggestions [35]. Other research also suggests that affective occupational commitment serves as a psychological driver of employees’ innovative behaviors [36]. Therefore, within the framework of this longitudinal study, in‐group/out‐group dynamics may indirectly influence nurses’ work improvement and innovation through affective occupational commitment.

Within organizational settings, an individual’s professional attitude is not determined solely by inherent personal characteristics but may also be gradually shaped and adjusted through ongoing social interactions [37]. Previous research has found that workplace social capital, as a resource‐based structure of social relationships, can enhance organizational cooperation and provide emotional support among nurses, thereby strengthening affective occupational commitment [38]. According to social information processing theory, high levels of workplace social capital may provide nurses with greater access to supportive interactions and informational resources within the organization. These social cues, through cognitive processing mechanisms, may reinforce nurses’ positive evaluations of professional values. Therefore, workplace social capital may contribute to affective occupational commitment by fostering more positive interpretations of the work environment and professional experiences.

Therefore, grounded in social information processing theory, this study aims to explore the mechanism through which in‐group/out‐group dynamics influence nurses’ work improvement and innovation via the chain mediation of workplace social capital and affective occupational commitment. However, Pérez‐Guerrero et al. noted that prior cross‐sectional studies capture only a snapshot at a single point in time [39], which constrains our understanding of the associations between in‐group/out‐group dynamics and work improvement and innovation. Consequently, by employing a three‐wave longitudinal design, this study intends to reveal the underlying mechanism through which environmental cues impact nurses’ improvement and innovation behaviors via organizational resources and professional attitudes. This design not only addresses the limited temporal information available from existing cross‐sectional research but also advances the research perspective toward processive explanations, more accurately capturing the processes of social information processing and attitude transformation.

The hypotheses for this study were as follows (Figure 1):

• Hypothesis 1. In‐group/out‐group dynamics has a direct effect on work improvement and innovation among nurses.
• Hypothesis 2. Workplace social capital mediates the relationship between in‐group/out‐group dynamics and work improvement and innovation among nurses.
• Hypothesis 3. Affective occupational commitment mediates the relationship between in‐group/out‐group dynamics and work improvement and innovation among nurses.
• Hypothesis 4. Workplace social capital and affective occupational commitment have a chain mediating effect on the relationship between in‐group/out‐group dynamics and work improvement and innovation among nurses.

**FIGURE 1:**
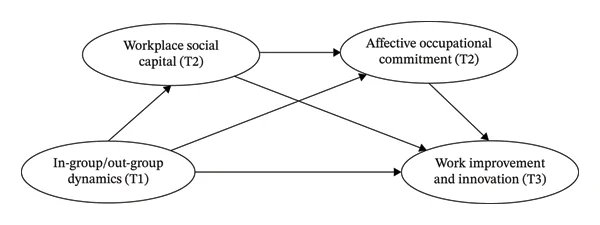
Hypothesized conceptual model. Notes: T1, Time 1; T2, Time 2; T3, Time 3.

## 2. Method

### 2.1. Study Design and Participants

A longitudinal study was conducted at Baoding Fourth Central Hospital in Hebei Province, China. Cluster sampling was conducted at the unit level. A total of 27 nursing units were included in the study, and all registered nurses within these units who met the eligibility criteria were invited to participate. These units covered diverse nursing practice settings, including internal medicine units (*n* = 9), surgical units (*n* = 9), gynecology (*n* = 1), pediatric department (*n* = 2), emergency department (*n* = 1), intensive care unit (*n* = 1), and procedural units (*n* = 4). Thus, the participating clusters varied in their clinical specialties and nursing practice contexts. The inclusion criteria were as follows: (1) being a registered nurse; (2) being currently employed by the hospital; and (3) having a minimum employment duration of 6 months. The exclusion criteria were as follows: (1) nurses who were absent from work during the survey period due to sick leave, maternity leave, or off‐site training; and (2) those currently participating in similar research projects.

Before the investigation commenced, the research team contacted the hospital’s nursing department to explain the objectives and significance of the study. After obtaining approval, the survey was formally initiated. The research team delivered the questionnaires to nursing managers via Questionnaire Star (https://www.wjx.cn/), an online survey platform widely utilized in China. Following the principles of voluntary participation and informed consent, nursing managers distributed the questionnaires to the nurses, each of which was accompanied by detailed instructions for completion. Throughout the study, participants were assigned unique codes to ensure the anonymity and traceability of all questionnaire data. Previous research has suggested that, in occupational health studies, a time lag of 3–6 months is typically sufficient to capture meaningful changes in most variables, and relatively short intervals can provide crucial insights into the evolution of psychological processes over time [40]. Therefore, data were collected in three waves at approximately 3‐month intervals: March 2024 (Time 1, T1), June 13–July 12, 2024 (Time 2, T2), and October 2024 (Time 3, T3). At T1, participants’ demographic information and baseline measures of in‐group/out‐group dynamics, workplace social capital, affective occupational commitment, and work improvement and innovation were collected. At T2, workplace social capital and affective occupational commitment were reassessed. At T3, work improvement and innovation was remeasured. The study was reported in accordance with the Strengthening the Reporting of Observational Studies in Epidemiology (STROBE) statement.

The minimum required sample size was estimated using G∗Power 3.1 [41]. *F*‐tests were applied, and the linear multiple regression fixed model with *R* ^2^ deviation from zero was selected. For a multiple regression model with 10 predictors, assuming a medium effect size (*f* ^2^ = 0.15), a significance level of *α* = 0.05, and a power (1 − *β*) of 0.95, the calculated required sample size was 172 participants. In addition, following recommendations from previous literature [42], Soper’s online a priori sample size calculator for structural equation models was used [43]. Considering 7 observed variables and 2 latent variables in the model, and assuming an anticipated effect size of 0.30, a probability level of 0.05, and a desired statistical power of 0.80, the estimated minimum sample size required to test the hypothesized model was 138 participants. A total of 479 nurses were initially invited to participate, of whom 5 declined at T1. At T1, 474 participants submitted complete questionnaires. At T2, 461 participants completed the survey, corresponding to a response rate of 97.26%. At T3, 459 participants engaged, corresponding to a response rate of 96.84%. Response rates at T2 and T3 stratified by baseline demographic characteristics are presented in Table S1. After matching the data across the three survey waves, 23 participants were excluded because of incomplete questionnaires or missing data on study variables. The final analytic sample comprised 436 participants, accounting for 91.98% of the baseline sample. The participant flow diagram is shown in Figure 2. Furthermore, to further evaluate potential selection bias introduced by participant attrition and exclusion, baseline characteristics were compared between included and excluded participants. No statistically significant differences were observed (all *p* > 0.05) (Table S2).

**FIGURE 2:**
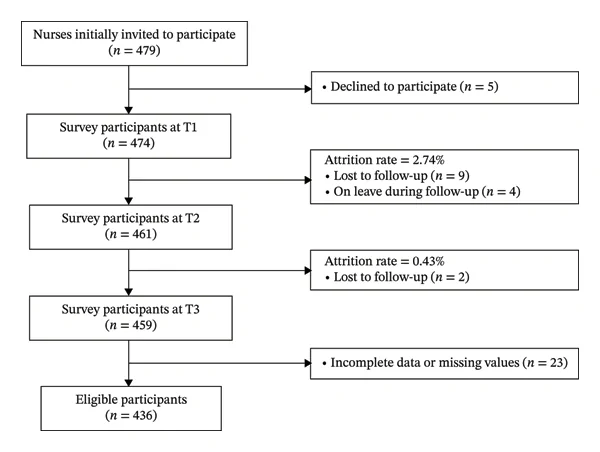
Flow diagram of participant selection. Notes: T1, Time 1; T2, Time 2; T3, Time 3.

### 2.2. Ethical Authorization

This study was conducted in accordance with the Declaration of Helsinki. Ethical approval was obtained from the Ethics Committee of Baoding Fourth Central Hospital (approval number: 2024007). All participants signed informed consent before enrollment and were granted the right to withdraw from the study at any moment without adverse consequences. This study was conducted anonymously to protect participants’ privacy. The data were accessible solely to the research team and used exclusively for statistical analysis.

### 2.3. Measures

#### 2.3.1. Sociodemographic Characteristics Questionnaire

Based on existing literature on the study variables [15, 44–47], this study developed a sociodemographic questionnaire to collect participants’ baseline characteristics. The sociodemographic data comprised age, gender (male, female), marital status (single, married), educational level (junior college or below, bachelor or above), type of employment (contract, permanent), professional title (junior, intermediate, deputy senior, senior), working years (≤ 5, 6∼10, 11∼15, 16∼20, ≥ 21), number of night shifts (per week) (0, 1, 2, ≥ 3), and monthly income (Chinese Yuan) (≤ 4000, 4001∼6000, ≥ 6001) (1 US dollar ≈ 7.18 Chinese Yuan in 2024).

#### 2.3.2. In‐Group/Out‐Group Dynamics

The 11‐item Chinese version of the in‐group/out‐group dynamics scale was utilized to evaluate in‐group/out‐group dynamics [48]. The scale has demonstrated good reliability and validity within the Chinese context [15]. Items were rated on a 5‐point Likert scale, ranging from 1 (*totally disagree*) to 5 (*totally agree*). The scale encompassed three dimensions: reciprocal attachment (six items), favoritism (three items), and having cronies (two items). The total score spanned from 11 to 55, with higher scores indicating higher levels of in‐group/out‐group dynamics. In the current study, Cronbach’s *α* of the scale was 0.909 at T1.

#### 2.3.3. Workplace Social Capital

The 8‐item of the workplace social capital scale was utilized to evaluate workplace social capital [49]. Items were rated on a 5‐point Likert scale, ranging from 1 (*totally disagree*) to 5 (*totally agree*). The scale encompassed two dimensions: cognitive dimension (three items) and structural dimension (five items). The total score spanned from 8 to 40, with higher scores indicating greater levels of workplace social capital. In the current study, Cronbach’s α of the scale was 0.964 at T1 and 0.973 at T2.

#### 2.3.4. Affective Occupational Commitment

The 6‐item of the affective occupational commitment scale was utilized to evaluate affective occupational commitment [50]. Items were rated on a 5‐point Likert scale, ranging from 1 (*totally disagree*) to 5 (*totally agree*). The total score spanned from 6 to 30, with higher scores indicating greater levels of affective occupational commitment. In the current study, Cronbach’s α of the scale was 0.964 at T1 and 0.972 at T2.

#### 2.3.5. Work Improvement and Innovation

The 6‐item of the work improvement and innovation scale was utilized to evaluate work improvement and innovation [51]. Items were rated on a 7‐point Likert scale, ranging from 1 (*totally disagree*) to 7 (*totally agree*). The total score spanned from 6 to 42, with higher scores indicating greater levels of work improvement and innovation. In the current study, Cronbach’s α of the scale was 0.806 at T1 and 0.798 at T3.

### 2.4. Data Analysis

Data analysis and model construction were performed using SPSS 29.0 (IBM Corp., NY, USA) and Mplus 8.0 software. Complete case analysis was used for the primary analyses, with missing data handled by listwise deletion. First, descriptive statistics were employed to summarize participants’ demographic characteristics, as well as their levels of in‐group/out‐group dynamics, workplace social capital, affective occupational commitment, and work improvement and innovation. The normality of continuous variables was assessed using the Kolmogorov–Smirnov test and visually inspected through Q‐Q plots, indicating that all continuous variables were approximately normally distributed. Thus, continuous variables were represented by means ± standard deviations, whereas categorical variables were presented as frequencies (percentages). Second, Pearson’s correlation analysis was used to examine the relationships between the study variables. Third, potential multicollinearity was assessed by calculating variance inflation factor (VIF) [52]. The results indicated no evidence of problematic multicollinearity among the independent variables (average VIF = 1.76, range 1.12−2.09). Fourth, confirmatory factor analysis (CFA) was conducted to evaluate the discriminant validity of the study constructs. Additionally, a common method factor was incorporated into the measurement model to examine potential common method bias [53]. Standardized factor loadings, composite reliability (CR), and average variance extracted (AVE) were also examined to assess the reliability and convergent validity of the measurement model. Fifth, structural equation modeling (SEM) used maximum likelihood (ML) estimation to examine the hypothesized pathways. Multidimensional constructs, including in‐group/out‐group dynamics and workplace social capital, were specified as latent variables, with their respective subdimensions serving as measurement indicators. Unidimensional constructs, including affective occupational commitment and work improvement and innovation, were represented as observed variables using total scores. Furthermore, following analytical recommendations from previous longitudinal mediation research [54, 55], baseline levels of the mediators and outcome variable were included in the model to control for autoregressive effects. Model fit was evaluated using multiple indices. Acceptable fit criteria included chi‐square (*χ* ^2^), degrees of freedom (df), chi‐square/degrees of freedom ratio (*χ* ^2^/df < 3.0), comparative fit index (CFI) and Tucker–Lewis index (TLI) both exceeding 0.90, and root mean square error of approximation (RMSEA) and standardized root mean square residual (SRMR) both below 0.08 [56]. The 95% confidence intervals (CIs) for the indirect effects were estimated using the bias‐corrected percentile bootstrap method (5000 resampling iterations) [57]. An indirect effect was considered statistically significant if its 95% CI did not include zero [58]. To further evaluate whether the sample size provided adequate statistical power to detect the indirect effects, a post hoc Monte Carlo power analysis with 10,000 replications was conducted [59]. Power values exceeding the threshold of 0.80 were considered adequate [60]. The simulation was implemented in Python 3.14.6 using the NumPy library. Sixth, based on previous literature [47, 61, 62], age, gender, education level, working years, professional title, weekly night shifts, and monthly income were incorporated as covariates to account for potential confounding. Additionally, considering that the actual time intervals between waves varied across participants, this study further conducted a sensitivity analysis by including the individual‐level T1–T2 and T2–T3 intervals as covariates in the chain mediation model. The T1–T2 interval was controlled when predicting the mediating variables (T2), and the T2–T3 interval was controlled when predicting the outcome variable (T3). All statistical tests were two‐tailed, with statistical significance set at *p* < 0.05.

## 3. Results

### 3.1. Sociodemographic Characteristics of Participants

The sociodemographic characteristics of the participants are presented in Table 1. Participants ranged in age from 21 to 60 years, with a mean age of 32.66 years (SD = 6.75). The majority were female, comprising 423 (97.02%) individuals. Among the participants, 326 (74.77%) were married and 298 (68.35%) held a bachelor’s degree or above. Over half, 256 (58.72%) were in contractual employment, 221 (50.69%) possessed junior professional titles, and 239 (54.82%) had at least 10 years of work experience. In addition, 361 (82.80%) individuals worked two or fewer night shifts per week. A monthly income of 4000 Yuan or less was reported by 213 (48.85%) participants (approximately 557 US dollars in 2024).

**TABLE 1:** Sociodemographic characteristics of participants (*N* = 436).

Variables	*n* (%)
Age (years)	32.66 ± 6.75 (mean ± SD)
Gender	
Male	13 (2.98)
Female	423 (97.02)
Marital status	
Single	110 (25.23)
Married	326 (74.77)
Education level	
Junior college or below	138 (31.65)
Bachelor or above	298 (68.35)
Type of employment	
Contract	256 (58.72)
Permanent	180 (41.28)
Professional title	
Junior	221 (50.69)
Intermediate	163 (37.39)
Deputy senior	43 (9.86)
Senior	9 (2.06)
Working years	
≤ 5	126 (28.90)
6∼10	71 (16.28)
11∼15	157 (36.01)
16∼20	44 (10.09)
≥ 21	38 (8.72)
Number of night shifts (per week)	
0	122 (27.98)
1	123 (28.21)
2	116 (26.61)
≥ 3	75 (17.20)
Monthly income (Chinese Yuan)	
≤ 4000	213 (48.85)
4001∼6000	184 (42.20)
≥ 6001	39 (8.94)

*Note:* Percentages might not add up to 100% due to rounding.

Abbreviation: SD, standard deviation.

### 3.2. Descriptive Statistics and Correlations Among Study Variables

Table 2 presents the means and standard deviations of the study variables. The total scores for in‐group/out‐group dynamics (T1), workplace social capital (T2), affective occupational commitment (T2), and work improvement and innovation (T3) among nurses were 34.06 ± 7.31 (score index using the actual score divided by the maximum attainable score, 61.93%), 33.31 ± 5.23 (score index, 83.28%), 24.71 ± 4.33 (score index, 82.37%), and 32.27 ± 5.24 (score index, 76.83%), respectively.

**TABLE 2:** Descriptive statistics and correlation analysis of study variables (*r*, *N* = 436).

Variables	Mean ± SD	1	2	3	4
1. In‐group/out‐group dynamics (T1)	34.06 ± 7.31	1			
2. Workplace social capital (T2)	33.31 ± 5.23	−0.300^∗∗∗^	1		
3. Affective occupational commitment (T2)	24.71 ± 4.33	−0.314^∗∗∗^	0.714^∗∗∗^	1	
4. Work improvement and innovation (T3)	32.27 ± 5.24	−0.253^∗∗∗^	0.414^∗∗∗^	0.418^∗∗∗^	1

*Note:* T1, Time 1; T2, Time 2; T3, Time 3.

Abbreviation: SD, standard deviation.

^∗∗∗^ *p* < 0.001.

The results of the bivariate correlation analysis among the research variables are also shown in Table 2. In‐group/out‐group dynamics (T1) was significantly negatively correlated with workplace social capital (T2) (*r* = −0.300, *p* < 0.001), affective occupational commitment (T2) (*r* = −0.314, *p* < 0.001), and work improvement and innovation (T3) (*r* = −0.253, *p* < 0.001). Workplace social capital (T2) was significantly positively correlated with affective occupational commitment (T2) (*r* = 0.714, *p* < 0.001) and work improvement and innovation (T3) (*r* = 0.414, *p* < 0.001). Furthermore, affective occupational commitment (T2) exhibited a positive correlation with work improvement and innovation (T3) (*r* = 0.418, *p* < 0.001).

### 3.3. CFA

Table 3 displays the fit indices of the hypothesized four‐factor model alongside other competing models. The four‐factor model demonstrated an optimal fit (*χ* ^2^ = 1217.234, df = 428, *χ* ^2^/df = 2.844, CFI = 0.911, TLI = 0.904, RMSEA = 0.065, SRMR = 0.058), outperforming alternative models, thereby supporting the discriminant validity of the study constructs. As shown in Table 3, the inclusion of the common method factor did not exhibit improved fit and instead showed a slight decline in fit compared with the theoretical model. This result indicated that common method bias did not have a substantial influence on the study findings. Additionally, all measurement indicators loaded significantly on their corresponding constructs in the four‐factor CFA model. Detailed standardized factor loadings, CR, and AVE are presented in Table S3.

**TABLE 3:** Confirmatory factor analysis results.

Model	*χ* ^2^	df	*χ* ^2^/df	CFI	TLI	RMSEA	SRMR
Four‐factor model + CMF: IOD, WSC, AOC, WII, CMF	1602.982	408	3.929	0.866	0.847	0.082	0.473
Four‐factor model: IOD, WSC, AOC, WII	1217.234	428	2.844	0.911	0.904	0.065	0.058
Three‐factor model: IOD + WSC, AOC, WII	2466.200	431	5.722	0.771	0.753	0.104	0.143
Three‐factor model: IOD, WSC + AOC, WII	2960.117	431	6.868	0.716	0.693	0.116	0.076
Two‐factor model: IOD + WSC + AOC, WII	4154.138	433	9.594	0.582	0.551	0.140	0.147
One‐factor model: IOD + WSC + AOC + WII	5304.488	434	12.222	0.452	0.413	0.160	0.169

*Note: N* = 436.

Abbreviations: AOC, affective occupational commitment; CFI, comparative fit index; CMF, common method factor; IOD, in‐group/out‐group dynamics; RMSEA, root mean square error of approximation; SRMR, standardized root mean square residual; TLI, Tucker–Lewis index; WII, work improvement and innovation; WSC, workplace social capital.

### 3.4. Model Fit Analysis

A structural model was specified and evaluated, with in‐group/out‐group dynamics (T1) as the predictor variable and with work improvement and innovation (T3) as the outcome variable. Workplace social capital (T2) and affective occupational commitment (T2) were specified as mediators. In addition, the baseline levels of mediators and the outcome variable were included in the model to control for their prior levels and account for their temporal stability. Meanwhile, age, gender, education level, working years, professional title, weekly night shifts, and monthly income were included as covariates in the model. The model fit indices were as follows: χ^2^ = 249.245, df = 92, χ^2^/df = 2.709 (< 3.0), CFI = 0.923 (> 0.90), TLI = 0.916 (> 0.90), RMSEA = 0.063 (< 0.08), and SRMR = 0.061 (< 0.08).

### 3.5. Analysis of Mediation Effect and Hypothesis Path Testing

As shown in Table 4 and Figure 3, in‐group/out‐group dynamics (T1) was a significant negative predictor of work improvement and innovation (T3) (*β* = −0.166, *p* = 0.002), supporting Hypothesis 1. Further path analysis revealed that in‐group/out‐group dynamics (T1) significantly negatively predicted workplace social capital (T2) (*β* = −0.247, *p* < 0.001). Workplace social capital (T2) significantly positively predicted work improvement and innovation (T3) (*β* = 0.244, *p* < 0.001). Additionally, in‐group/out‐group dynamics (T1) significantly negatively predicted affective occupational commitment (T2) (*β* = −0.119, *p* = 0.001), whereas affective occupational commitment (T2) positively predicted work improvement and innovation (T3) (*β* = 0.167, *p* = 0.009). Moreover, a significant positive path was observed between workplace social capital (T2) and affective occupational commitment (T2) (*β* = 0.644, *p* < 0.001).

**TABLE 4:** Regression analysis of the relationship among study variables in the mediation model.

Outcome variable	Predictor variable	*R* ^2^	*β*	SE	*t*	95% CI	*p*
Workplace social capital (T2)	In‐group/out‐group dynamics (T1)	0.296	−0.247	0.047	−5.304	(−0.338, −0.151)	< 0.001

Affective occupational commitment (T2)	In‐group/out‐group dynamics (T1)	0.546	−0.119	0.037	−3.268	(−0.192, −0.048)	0.001
	Workplace social capital (T2)		0.644	0.043	14.821	(0.553,0.725)	< 0.001

Work improvement and innovation (T3)	In‐group/out‐group dynamics (T1)	0.268	−0.166	0.052	−3.173	(−0.266, −0.060)	0.002
	Workplace social capital (T2)		0.244	0.064	3.824	(0.121,0.365)	< 0.001
	Affective occupational commitment (T2)		0.167	0.064	2.606	(0.034,0.287)	0.009

*Note:* T1, Time 1; T2, Time 2; T3, Time 3. All paths are expressed as standardized regression coefficients. *β*, regression coefficients.

Abbreviations: CI, confidence interval; SE, standard error.

**FIGURE 3:**
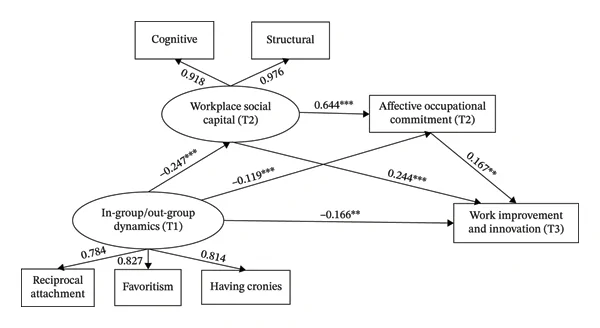
The chain model of the mediating effect of workplace social capital (T2) and affective occupational commitment (T2) between in‐group/out‐group dynamics (T1) and work improvement and innovation (T3). Notes: T1, Time 1; T2, Time 2; T3, Time 3. Age, gender, education level, working years, professional title, weekly night shifts, and monthly income were treated as covariates in the chain mediation model. All paths are expressed as standardized regression coefficients. ^∗∗^ *p* < 0.01, ^∗∗∗^ *p* < 0.001.

The results of the chain mediation pathway analysis are presented in Table 5. Both direct and indirect effects were observed between in‐group/out‐group dynamics and work improvement and innovation. The total indirect effect was −0.107 (95% CI [−0.154, −0.069]), accounting for 39.19% of the total effect. Three specific indirect pathways were identified. For Path 1, the indirect effect of in‐group/out‐group dynamics (T1) on work improvement and innovation (T3) through workplace social capital (T2) was −0.060 (95% CI [−0.106, −0.029]), supporting Hypothesis 2. For Path 2, the indirect effect of in‐group/out‐group dynamics (T1) on work improvement and innovation (T3) through affective occupational commitment (T2) was −0.020 (95% CI [−0.046, −0.005]), supporting Hypothesis 3. For Path 3, the chain indirect effect of in‐group/out‐group dynamics (T1) on work improvement and innovation (T3) through workplace social capital (T2) and affective occupational commitment (T2) was −0.027 (95% CI [−0.052, −0.007]), supporting Hypothesis 4.

**TABLE 5:** Total, direct, and indirect effects of the hypothesized mediation model.

Pathway	Effect	Boot SE	95% CI	*p*
Total effect	−0.273	0.049	(−0.364, −0.168)	< 0.001
Direct effect	−0.166	0.052	(−0.266, −0.060)	0.002
Total indirect effect	−0.107	0.022	(−0.154, −0.069)	< 0.001
Indirect 1: In‐group/out‐group dynamics (T1) ⟶ Workplace social capital (T2) ⟶ Work improvement and innovation (T3)	−0.060	0.019	(−0.106, −0.029)	0.002
Indirect 2: In‐group/out‐group dynamics (T1) ⟶ Affective occupational commitment (T2) ⟶ Work improvement and innovation (T3)	−0.020	0.010	(−0.046, −0.005)	0.046
Indirect 3: In‐group/out‐group dynamics (T1) ⟶ Workplace social capital (T2) ⟶ Affective occupational commitment (T2) ⟶ Work improvement and innovation (T3)	−0.027	0.011	(−0.052, −0.007)	0.019

*Note:* T1, Time 1; T2, Time 2; T3, Time 3. All paths are expressed as standardized regression coefficients.

Abbreviations: Boot SE, bootstrapping standard error; CI, confidence interval.

The post hoc Monte Carlo power analysis yielded estimated power values of 0.947 for the indirect effect through workplace social capital, 0.834 for the indirect effect through affective occupational commitment, and 0.953 for the chained indirect effect through workplace social capital and affective occupational commitment. These results indicated adequate statistical power for the model.

### 3.6. Sensitivity Analysis

After further controlling for the individual‐level time intervals, the direction and statistical significance of the pathways remained consistent with the original model. These results indicated that the differential exposure durations across participants did not substantially affect the main findings of this study. Detailed results are presented in Table S4.

## 4. Discussion

To our knowledge, this is the first study to investigate the mediating roles of workplace social capital (T2) and affective occupational commitment (T2) in the relationship between in‐group/out‐group dynamics (T1) and work improvement and innovation (T3) among Chinese nurses. The findings showed that in‐group/out‐group dynamics (T1) was significantly negatively associated with subsequent work improvement and innovation (T3). Furthermore, both workplace social capital (T2) and affective occupational commitment (T2) functioned as chain mediators in this relationship.

This study indicated that in‐group/out‐group dynamics (T1) was significantly negatively associated with nurses’ work improvement and innovation (T3). A cross‐sectional study of 283 Pakistani nurses found that positive diversity climate was significantly positively related to employees’ innovative work behaviors [63]. This study extends these findings by showing that in‐group/out‐group dynamics, reflecting perceptions of a negative work climate, was prospectively linked to nurses’ work improvement and innovation 6 months later. Power distance within organizations may provide a cultural contextual explanation for understanding the negative association between in‐group/out‐group dynamics and work improvement and innovation among nurses. Chinese society exemplifies a high‐power distance culture, emphasizing hierarchical order and clear distinctions between superiors and subordinates [64]. In‐group/out‐group dynamics characterized by a leader‐centered relational network and tiered differentiated treatment based on the proximity of relationships [65] may represent an organizational manifestation of this cultural context. In healthcare organizations, rigid hierarchical structures have been associated with a lower willingness among employees to proactively propose innovations or question existing workflows [66]. Simultaneously, staff tend to prioritize maintaining interpersonal harmony during interactions rather than driving change or challenging the status quo [67]. In this context, nurses may be more inclined to conform to established norms, potentially limiting devotion to work improvement and innovation. Additionally, according to social information processing theory, individuals interpret social information within organizational contexts and adjust their behavior accordingly [19]. From this theoretical perspective, differential treatment by leaders based on relational closeness may constitute an important social cue, enabling employees to infer their relative position within the organization’s relational network. Managers may categorize subordinates as “insiders” or “outsiders” based on the closeness of their interactions, subsequently allocating resources accordingly [68]. Members who are closer to core leaders are generally afforded greater privileges and additional organizational resources [16]. Conversely, employees who perceive themselves as belonging to an out‐group are prone to perceiving inequities in resource allocation, which may correspond to lower intrinsic motivation for innovation [69]. Therefore, nurse managers should allocate work‐related information and organizational resources according to transparent and consistent criteria [70], thereby reducing disparities in opportunities arising from interpersonal closeness. Establishing clear feedback mechanisms and actively soliciting and valuing nurses’ suggestions for improvement may help strengthen psychological safety and promote voice behavior [71]. Hospitals may also hold unit‐level shared governance meetings to provide nurses with formal avenues to participate in nursing decision‐making and quality improvement initiatives [72], thereby creating more equitable conditions for proposing and implementing work improvement and innovation.

This study found that workplace social capital (T2) mediated the relationship between in‐group/out‐group dynamics (T1) and work improvement and innovation (T3). A previous study involving 387 employees identified social capital as a vital resource associated with innovative behavior [73], which aligns with our findings. Notably, this study further extends the argument that workplace social capital was not only directly related to work improvement and innovation, but also accounted for an indirect pathway linking in‐group/out‐group dynamics to work improvement and innovation. In‐group/out‐group dynamics prospectively predicted workplace social capital approximately 3 months later, which was subsequently related to work improvement and innovation 6 months thereafter. On the one hand, in‐group/out‐group dynamics undermine the foundation of trust among colleagues, thereby eroding workplace social capital. Within nursing teams, social capital is closely grounded in mutual trust among team members, confidence in managerial fairness, and shared values [74]. In‐group/out‐group dynamics are often characterized by relationship‐oriented and informal patterns of resource allocation [75], which may weaken established foundations of trust and shared value within the organization and, in turn, be associated with lower workplace social capital [28]. Notably, in‐group/out‐group dynamics may not simply weaken trust but may instead make trust more closely tied to specific members and relational circles [76]. For nursing teams, this relationship‐specific pattern of trust may further constrain the development of workplace social capital at the team level. On the other hand, workplace social capital may provide favorable conditions for knowledge exchange and reorganization, serving as a crucial driver of innovation. According to social network theory, social networks comprise complex webs of interconnected interpersonal relationships [77]. Higher levels of workplace social capital have been linked to more cohesive networks among members, facilitating efficient work performance [78]. Additionally, workplace social capital is related to greater trust and resource sharing, which may correspond to information‐sharing willingness and collaborative efficiency, ultimately driving work improvement and innovation [79, 80]. Nurse managers should promote collaboration and connections between nurses situated within and outside relational circles. For example, nurses recognized by their peers could be considered as social network brokers to facilitate information exchange across different relational circles during shift handovers, case discussions, and nursing improvement activities [81]. Strengthening team collaboration mechanisms may help develop stable interpersonal networks and, in turn, support workplace social capital [82]. Furthermore, cultivating an open organizational communication environment may encourage knowledge sharing and reciprocal exchange, providing supportive conditions for nurses’ work improvement and innovation [83].

Our findings suggested that affective occupational commitment (T2) mediated the relationship between in‐group/out‐group dynamics (T1) and work improvement and innovation (T3) among nurses. A cross‐sectional study involving 321 nurses found that affective occupational commitment positively predicts innovative behaviors in the workplace [84], which is consistent with our view. However, this study further elucidated a potential pathway from organizational climate to attitude to behavior, revealing that in‐group/out‐group dynamics was prospectively related to nurses’ affective occupational commitment, which in turn was linked to subsequent work improvement and innovation. The relationship between in‐group/out‐group dynamics and affective occupational commitment may be understood through social exchange theory. This theory posits that employees determine the quality of their social exchange relationships by evaluating the fairness between personal contributions and organizational reciprocation [85]. In the relationship‐oriented organizational context of China, in‐group/out‐group dynamics may reflect not only disparities in resources and benefits but also social cues regarding whether nurses are recognized and valued by the organization. When nurses perceive unfairness or relationship‐oriented differential treatment, they may report lower organizational trust and emotional investment, which may in turn be associated with weaker affective occupational commitment [86]. Moreover, affective occupational commitment reflects an individual’s emotional identification with their profession and sense of belonging, serving as a crucial psychological foundation for work improvement and innovation. Within the Chinese cultural context, the benevolence of Confucianism is conceptually aligned with the altruistic moral character inherent in the nursing profession [87]. Such cultural congruence may lend greater cultural salience to affective occupational commitment among Chinese nurses. Higher levels of affective occupational commitment have been linked to a stronger sense of professional belonging [88], greater engagement in professional development, and more proactive identification of issues in clinical practice, which may promote work improvement and innovation [84, 89]. Nurse managers could consider establishing transparent and equitable recognition and incentive mechanisms centered on professional contributions, rather than informal management driven by interpersonal preferences. Timely and specific feedback, together with appropriate formal recognition [90], could be provided based on nurses’ actual performance in clinical care and peer collaboration. This approach may help avoid the disproportionate concentration of recognition and incentives among a few members with close relationships with leaders. Previous research has shown that fostering positive leadership behaviors, such as providing regular feedback and acknowledging achievements, significantly enhances nurses’ affective occupational commitment [91]. Meanwhile, cultivating a work environment supportive of professional growth by providing continuing professional development resources and integrating innovative practices into career development evaluation systems [92, 93], which can help stimulate nurses’ sustained engagement in work improvement and innovation.

Research findings showed a significant chain indirect pathway linking in‐group/out‐group dynamics (T1) to work improvement and innovation (T3) through workplace social capital (T2) and affective occupational commitment (T2). A previous cross‐sectional study involving 1220 nurses indicated that higher workplace social capital was positively related to affective occupational commitment [94]. However, our findings deepen the understanding of the underlying mechanisms of in‐group/out‐group dynamics by highlighting the roles of workplace social capital and affective occupational commitment as essential psychological resources associated with work improvement and innovation. This study therefore provides a more comprehensive perspective for comprehending the development of work improvement and innovation. Workplace social capital is a vital element of a supportive and trustworthy healthy work environment [79]. It offers nurses stable positive social information through trust, reciprocity, and collaborative relationships [45]. Through the interpretation of these cues, nurses may develop more positive evaluations of their organizational and professional identities, which may constitute a crucial psychological basis for affective occupational commitment [95]. Moreover, under the culturally dominant framework of Chinese collectivism, nurses typically place a high value on team belonging and organizational recognition [96]. From this cultural perspective, relational resources embodied in workplace social capital, including trust, reciprocity, and a sense of belonging, may be particularly meaningful in promoting affective occupational commitment among Chinese nurses. Managers should implement cross‐departmental collaboration and information‐sharing mechanisms, facilitating semiformal communication opportunities for nurses to express their professional values and ideas, thereby supporting relational networks among nurses and workplace social capital [97, 98]. Such approaches also provide emotional and resource support, reinforcing nurses’ affective occupational commitment [94]. These can help establish a solid psychological resource foundation to counteract the potential negative impact of in‐group/out‐group dynamics, serving protective buffering effects on work improvement and innovation to some extent.

Notably, the findings of this study may possess a degree of Chinese cultural embeddedness. In‐group/out‐group dynamics can be understood as an organizational extension of a high‐power distance culture, in which in‐group/out‐group distinctions involve not only formal work exchanges but may also be influenced by affective proximity, private interactions, and compliance with leadership authority. In such a relationship‐oriented organizational environment, resources may be disproportionately concentrated among insiders, potentially creating barriers to information and support between in‐group and out‐group members. Meanwhile, maintaining interpersonal harmony and face‐saving are deeply rooted in Chinese culture and the healthcare system [99]. These cultural factors may make nurses more reluctant to openly question differential treatment and may instead be reflected in more covert responses, such as envy and silence [15]. It is noteworthy that this does not imply that Chinese culture inevitably leads to differential management; rather, it highlights that these cultural factors can potentially intensify the effects of in‐group/out‐group dynamics under specific organizational conditions. Consequently, future research should further examine the cultural boundaries of these relationships across different cultures and organizational contexts.

## 5. Limitations

This study has several limitations. First, the data were collected using self‐report questionnaires, which inherently involve a certain degree of subjectivity and may introduce response bias, particularly social desirability bias. This concern may be especially relevant to socially valued workplace behaviors, such as work improvement and innovation, because participants may have provided overly favorable assessments of their own performance. Future research should consider employing multisource measurement approaches by incorporating performance indicators or supervisor ratings to enhance objectivity and validity of measurement. Second, the sample for this study was drawn from a single hospital in China, which may restrict the generalizability of the findings. The variables investigated are embedded within organizational, managerial, and cultural contexts and may therefore be influenced by institutional characteristics and contextual factors. Future research should broaden the sampling scope through multicenter studies across healthcare institutions in different regions and conduct cross‐cultural comparisons to further examine these findings. Third, although this study was guided by theoretically derived hypotheses, conducted with institutional ethical approval, and implemented under an approved research project, the study protocol was not prospectively preregistered before data collection. The absence of preregistration may limit transparency regarding the original research plan and analytical decisions. Future studies are encouraged to consider prospective preregistration to further enhance methodological transparency. Fourth, the potential impact of unmeasured temporal confounders during the follow‐up period cannot be fully ruled out. These may include events such as adjustments in hospital policies, leadership turnover, variations in staffing or workload, and public health emergencies. Future research should concurrently document significant organizational changes during the follow‐up period and consider incorporating relevant contextual variables to enhance the interpretability and robustness of the findings. Fifth, although the three‐wave longitudinal design allowed temporal separation between the predictor and outcome variables, workplace social capital and affective occupational commitment were assessed concurrently at T2. The temporal ordering between these two mediators could not be fully determined. Future studies employing multiple measurement points for each mediator are needed to further validate the proposed sequential pathway. Sixth, although the longitudinal design and adjustment for baseline levels strengthen temporal inference, the possibility of reverse or reciprocal relationships cannot be completely excluded. For example, nurses with greater work improvement and innovation may develop stronger workplace social capital. This bidirectionality limits causal inferences. Future studies could employ experimental and intensive longitudinal designs to further examine the proposed mechanisms.

## 6. Conclusion

Our findings indicate that for nurses, in‐group/out‐group dynamics was negatively associated with work improvement and innovation, with the chain mediating role of workplace social capital and affective occupational commitment in this relationship. This study provides empirical evidence with implications for nursing management practice. Nursing managers should focus on institutional fairness, team trust, and occupational emotional support to improve the organizational atmosphere, which may help facilitate sustained development of improvement and innovation behaviors among nurses. Future research should incorporate multicenter data and evaluate context‐specific interventions to further examine the long‐term implications of in‐group/out‐group dynamics on work improvement and innovation, as well as identify targeted intervention strategies.

## Author Contributions

Chen Huang and Yu Yang share the first authorship of this work. Chen Huang: formal analysis, conceptualization, methodology, software, writing–original draft. Yu Yang: conceptualization, writing–review and editing. Yifan Wu: writing–review and editing. Zuoming Zhang: writing–review and editing. Hongmei Liu: data collection, writing–review and editing. Lin Cai: data collection, investigation, writing–review and editing, supervision. Shuang Zang: conceptualization, writing–review and editing, supervision.

## Funding

This work was supported by Baoding Science and Technology Plan Project (No. 2441ZF189).

## Conflicts of Interest

The authors declare no conflicts of interest.

## Supporting Information

Additional supporting information can be found online in the Supporting Information section.

## Supporting information


**Supporting Information** Table S1: Response rates at T2 and T3 stratified by baseline demographic characteristics. Table S2: Comparison of baseline characteristics of included and excluded participants from the longitudinal study. Table S3: Standardized factor loadings for the four‐factor confirmatory factor analysis model. Table S4: Sensitivity analysis controlling for individual‐level time intervals between phases.

## Data Availability

The data that support the findings of this study are available from the corresponding author upon reasonable request.
