# Supplementary material for: The Mediating Role of Workplace Social Capital and Affective Occupational Commitment in the Relationship Between In‐Group/Out‐Group Dynamics and Work Improvement and Innovation Among Nurses: A Longitudinal Study
Source: J Nurs Manag. 2026 Oct 22;2026:4125257. doi: 10.1155/jonm/4125257 (PMC13691289; doi:10.1155/jonm/4125257)
Supplement: Supplementary file 1 [file JONM-2026-4125257-s001.docx]

***Supplementary material***

Table S1 Response rates at T2 and T3 stratified by baseline demographic characteristics.

Table S2 Comparison of baseline characteristics of included and excluded participants from the longitudinal study.

Table S3 Standardized factor loadings for the four-factor confirmatory factor analysis model.

Table S4 Sensitivity analysis controlling for individual-level time intervals between phases.

**Table S1** Response rates at T2 and T3 stratified by baseline demographic characteristics.

| **Variables** | **T1 baseline sample, n** | **T2 response, n (%)** | **T3 response, n (%)** |
| --- | --- | --- | --- |
| Age (years) |  |  |  |
| ≤ 25 | 30 | 29 (96.67) | 28 (93.33) |
| 26~35 | 217 | 208 (95.85) | 207 (95.39) |
| 36~45 | 203 | 200 (98.52) | 200 (98.52) |
| ≥ 46 | 24 | 24 (100.00) | 24 (100.00) |
| Gender |  |  |  |
| Male | 16 | 15 (93.75) | 15 (93.75) |
| Female | 458 | 446 (97.38) | 444 (96.94) |
| Marital status |  |  |  |
| Single | 117 | 115 (98.29) | 115 (98.29) |
| Married | 357 | 346 (96.92) | 344 (96.36) |
| Education level |  |  |  |
| Junior college or below | 149 | 145 (97.32) | 143 (95.97) |
| Bachelor or above | 325 | 316 (97.23) | 316 (97.23) |
| Type of employment |  |  |  |
| Contract | 280 | 271 (96.79) | 269 (96.07) |
| Permanent | 194 | 190 (97.94) | 190 (97.94) |
| Professional title |  |  |  |
| Junior | 241 | 233 (96.68) | 231(95.85) |
| Intermediate | 179 | 174 (97.21) | 174 (97.21) |
| Deputy senior | 44 | 44 (100.00) | 44 (100.00) |
| Senior | 10 | 10 (100.00) | 10 (100.00) |
| Working years |  |  |  |
| ≤ 5 | 135 | 132 (97.78) | 130 (96.30) |
| 6~10 | 81 | 79 (97.53) | 79 (97.53) |
| 11~15 | 173 | 166 (95.95) | 166 (95.95) |
| 16~20 | 45 | 45 (100.00) | 45 (100.00) |
| ≥ 21 | 40 | 39 (97.50) | 39 (97.50) |
| Number of night shifts (per week) |  |  |  |
| 0 | 134 | 129 (96.27) | 127 (94.78) |
| 1 | 131 | 129 (98.47) | 129 (98.47) |
| 2 | 127 | 123 (96.85) | 123 (96.85) |
| ≥ 3 | 82 | 80 (97.56) | 80 (97.56) |
| Monthly income (Chinese Yuan) |  |  |  |
| ≤ 4000 | 236 | 227 (96.19) | 225 (95.34) |
| 4001~6000 | 195 | 192 (98.46) | 192 (98.46) |
| ≥ 6001 | 43 | 42 (97.67) | 42 (97.67) |

Notes: T1, time 1; T2, time 2; T3, time 3.

**Table S2** Comparison of baseline characteristics of included and excluded participants from the longitudinal study.

| **Variables** | **Included participants**  **(n = 436)** | **Excluded participants**  **(n = 38)** | ***p*** |
| --- | --- | --- | --- |
| **Continuous variable, (Mean ± SD)** |  |  |  |
| Age (years) | 32.66 **±** 6.75 | 31.68 **±** 5.51 | 0.389 |
| **Categorical variable, n (%)** |  |  |  |
| Gender |  |  | 0.129 |
| Male | 13 (2.98) | 3 (7.89) |  |
| Female | 423 (97.02) | 35 (92.11) |  |
| Marital status |  |  | 0.351 |
| Single | 110 (25.23) | 7 (18.42) |  |
| Married | 326 (74.77) | 31(81.58) |  |
| Education level |  |  | 0.731 |
| Junior college or below | 138 (31.65) | 11 (28.95) |  |
| Bachelor or above | 298 (68.35) | 27(71.05) |  |
| Type of employment |  |  | 0.532 |
| Contract | 256 (58.72) | 24 (63.16) |  |
| Permanent | 180 (41.28) | 14 (36.84) |  |
| Professional title |  |  | 0.521 |
| Junior | 221 (50.69) | 20 (52.63) |  |
| Intermediate | 163 (37.39) | 16 (42.11) |  |
| Deputy senior | 43 (9.86) | 1 (2.63) |  |
| Senior | 9 (2.06) | 1 (2.63) |  |
| Working years |  |  | 0.259 |
| ≤ 5 | 126 (28.90) | 9 (23.68) |  |
| 6~10 | 71 (16.28) | 10 (26.32) |  |
| 11~15 | 157 (36.01) | 16 (42.11) |  |
| 16~20 | 44 (10.09) | 1 (2.63) |  |
| ≥ 21 | 38 (8.72) | 2 (5.26) |  |
| Number of night shifts (per week) |  |  | 0.823 |
| 0 | 122 (27.98) | 12 (31.58) |  |
| 1 | 123 (28.21) | 8 (21.05) |  |
| 2 | 116 (26.61) | 11 (28.95) |  |
| ≥ 3 | 75 (17.20) | 7 (18.42) |  |
| Monthly income (Chinese Yuan) |  |  | 0.280 |
| ≤ 4000 | 213 (48.85) | 23 (60.53) |  |
| 4001~6000 | 184 (42.20) | 11 (28.95) |  |
| ≥ 6001 | 39 (8.94) | 4 (10.53) |  |

Note: Percentages might not add up to 100% due to rounding.

Abbreviation: SD, standard deviation.

**Table S3** Standardized factor loadings for the four-factor confirmatory factor analysis model.

| **Construct** | **Measurement indicator** | **Standardized factor loading** | **SE** | ***p*** | **CR** | **AVE** |
| --- | --- | --- | --- | --- | --- | --- |
| IOD |  |  |  |  | 0.851 | 0.656 |
|  | Reciprocal attachment | 0.793 | 0.033 | <0.001 |  |  |
|  | Favoritism | 0.817 | 0.033 | <0.001 |  |  |
|  | Having cronies | 0.819 | 0.031 | <0.001 |  |  |
| WSC |  |  |  |  | 0.945 | 0.897 |
|  | Cognitive | 0.921 | 0.017 | <0.001 |  |  |
|  | Structural | 0.972 | 0.013 | <0.001 |  |  |
| AOC |  |  |  |  | 0.974 | 0.861 |
|  | AOC 1 | 0.748 | 0.032 | <0.001 |  |  |
|  | AOC 2 | 0.951 | 0.011 | <0.001 |  |  |
|  | AOC 3 | 0.965 | 0.007 | <0.001 |  |  |
|  | AOC 4 | 0.982 | 0.004 | <0.001 |  |  |
|  | AOC 5 | 0.961 | 0.008 | <0.001 |  |  |
|  | AOC 6 | 0.941 | 0.012 | <0.001 |  |  |
| WII |  |  |  |  | 0.965 | 0.820 |
|  | WII 1 | 0.889 | 0.030 | <0.001 |  |  |
|  | WII 2 | 0.925 | 0.024 | <0.001 |  |  |
|  | WII 3 | 0.967 | 0.010 | <0.001 |  |  |
|  | WII 4 | 0.905 | 0.025 | <0.001 |  |  |
|  | WII 5 | 0.891 | 0.024 | <0.001 |  |  |
|  | WII 6 | 0.853 | 0.035 | <0.001 |  |  |

Notes: For IOD and WSC, sub-dimension scores were used as measurement indicators; for AOC and WII, individual items were used as measurement indicators.

Abbreviations: IOD, in-group/out-group dynamics; WSC, workplace social capital; AOC, affective occupational commitment; WII, work improvement and innovation; SE, standard error; CR, composite reliability; AVE, average variance extracted.

**Table S4** Sensitivity analysis controlling for individual-level time intervals between phases.

| **Pathway** | **Effect** | **Boot SE** | **95% CI** | ***P*** |
| --- | --- | --- | --- | --- |
| Total effect | -0.272 | 0.049 | (-0.364, -0.169) | <0.001 |
| Direct effect | -0.164 | 0.052 | (-0.264, -0.060) | 0.002 |
| Total indirect effect | -0.108 | 0.022 | (-0.155, -0.071) | <0.001 |
| Indirect 1: In-group/out-group dynamics (T1) ⟶ Workplace social capital (T2) ⟶ Work improvement and innovation (T3) | -0.059 | 0.019 | (-0.104, -0.028) | 0.002 |
| Indirect 2: In-group/out-group dynamics (T1) ⟶ Affective occupational commitment (T2) ⟶ Work improvement and innovation (T3) | -0.021 | 0.010 | (-0.046, -0.006) | 0.037 |
| Indirect 3: In-group/out-group dynamics (T1) ⟶ Workplace social capital (T2) ⟶ Affective occupational commitment (T2) ⟶ Work improvement and innovation (T3) | -0.028 | 0.011 | (-0.053, -0.009) | 0.015 |

Notes: T1, time 1; T2, time 2; T3, time 3. All paths are expressed as standardized regression coefficients.

Abbreviations: Boot SE, bootstrapping standard error; CI, confidence interval.
